# Fish Behavior as a Neural Proxy to Reveal Physiological States

**DOI:** 10.3389/fphys.2022.937432

**Published:** 2022-07-13

**Authors:** Chih-Wei Fu, Jiun-Lin Horng, Ming-Yi Chou

**Affiliations:** ^1^ Department of Life Science, National Taiwan University, Taipei, Taiwan; ^2^ Department of Anatomy and Cell Biology, School of Medicine, College of Medicine, Taipei Medical University, Taipei, Taiwan

**Keywords:** behaviors, physiology, environmental acclimation, neural circuits, toxicology

## Abstract

Behaviors are the integrative outcomes of the nervous system, which senses and responds to the internal physiological status and external stimuli. Teleosts are aquatic organisms which are more easily affected by the surrounding environment compared to terrestrial animals. To date, behavioral tests have been widely used to assess potential environmental risks using fish as model animals. In this review, we summarized recent studies regarding the effects of internal and external stimuli on fish behaviors. We concluded that behaviors reflect environmental and physiological changes, which have possible implications for environmental and physiological assessments.

## Introduction

Maintaining stable internal conditions in the face of environmental fluctuations is important for animals to sustain life. Consequently, animals have evolved strategies to respond and acclimate to environmental changes. The large time scale of acclimation such as morphology, to be selected over generations to fit the animal into the specific niches. With the increase of acclimated flexibility, acclimation happens also in a relatively short time. Physiological and behavioral acclimation provides the mid-term and short-term acclimation, respectively, to confront the changing environments. Revealing the acclimation axis across physiology and behavior could not only delineate a more comprehensive picture of how a niche is determined for the animal but also raise the opportunity to pose the assumption in envisioning how the brain works to coordinate the body as a whole. However, compared to morphology, acclimated physiology and behavior are mostly hidden without a specific design of examination. In addition to the diversity in morphology, the physiological and behavioral diversities could show us the plasticity of brain circuit and abundance of designs for acclimating to the dynamic aquatic environments (Cooper et al., 2013; Salvanes et al., 2013; Sewall 2015; Tseng et al., 2020). The acclimation in a relatively short time scale could be seen as the balance of maneuver (external behavior) and talent (internal physiological acclimation) (Staddon, 2016).

Fish lives in aquatic habits and are more easily affected by the surrounding environments with diverse ion compositions and anthropogenic pollution compared to terrestrial animals (Hwang and Lee, 2007; Busch et al., 2016). Therefore, fish were chosen as animal models for environmental assessments (Scholz et al., 2008; Green and Planchart, 2018). In this review, we summarized the acclimation strategies of behavior and internal autonomous regulation under the several states of homeostasis in teleost. We discussed how the internal regulation co-opts the behavior in acclimation with a general view and extend it to the current understandings of the applications in environmental toxicology.

## Effects of the Internal Status on Behaviors

Animals tend to maintain conditions that are best for their survival *via* physiological regulation, which is called “homeostasis”. Behaviors are integrative outcomes and rapidly respond to physiological changes for homeostasis. Alteration of physiological status constantly leads to behavioral response. Therefore, the process of physiological regulation can cause behavioral changes.

### Effects of Physiological Conditions on Fish Behaviors

Body fluid regulation is essential for organism survival. Hyperosmolality of body fluids activates osmotic-sensing neurons in the vascular organ of the lamina terminalis (VOLT) and subfornical organ (SFO), leading to vasopressin release and a drinking response (Bazyan and Rogal, 2015; Abbott et al., 2016). Recent research indicated that the terminus of drinking behavior is not directly regulated by changes in blood osmolality (Kim et al., 2021). Water intake rapidly reduces the activity of glutamatergic neurons in the SFO to baseline activity and vasopressin levels in the plasma before plasma osmolality recovers, thus preventing hypo-osmolality (Mandelblat-Cerf et al., 2017). Unlike terrestrial animals, most fish can drink surrounding water by a swallowing reflex without inducing a feeling of thirst or water-seeking behaviors. Mudskippers, a kind of amphibian teleost, can use buccal water for gas exchange when on land. Buccal drying motivates them move to water and drink, as occurs with tetrapods, through regulating angiotensin II and vasopressin (Katayama et al., 2018). This suggests a highly conserved osmoregulatory mechanism between teleost fishes and mammals. This case shows nervous system integrate physiological conditions and mediate behavioral outcomes to maintain homeostasis, including in early vertebrates.

Nutrients is necessary for life; thus, animal may be more proactive for survival when limited or depleted nutrients. A shortage of food drives animals to exhibit risky foraging behaviors (Katz et al., 2013; Padilla et al., 2016; Balaban-Feld et al., 2019). Hungry goldfish (*Carassius auratus*) left a group of conspecifics and shelter for foraging opportunities in the presence of a predator (Balaban-Feld et al., 2019). Agouti-related peptide (AgRP) neurons in the ventromedial part of the arcuate nucleus in the hypothalamus respond to energy deficits, activates feeding behavior and regulates metabolism (Belgardt et al., 2009; Berrios et al., 2021). In addition, AgRP expressed neurons send the projection to the brain regions implicated in aggression, fear and stress responses, which might modulate risky food seeking behaviors (Padilla et al., 2016; Shainer et al., 2019). Orexin/hypocretin, a neuropeptide secreted in the hypothalamus, regulates appetite and modulates fighting behavior that increase the winning rate of social conflicts after food deprivation (Kotz et al., 2002; Nakajo et al., 2020). Starvation increases orexin/hypocretin-positive neuron activity in the hypothalamus, which mediates neural plasticity in the dorsal interpeduncular nucleus (dIPN) and potentiates the winner’s pathway from the dorsal habenula to the dIPN (Nakajo et al., 2020). Taken together, animals take aggressive behavioral strategies to cope with starvation through the neuroendocrine system.

Behavior patterns during stress acclimation exhibited a property of energy reallocation to enhance behaviors that are more essential to survival. The Hypothalamic–pituitary–adrenal/interrenal (HPA/HPI) axis is main pathway to regulate physiological state and behaviors for coping with stress (Suarez-Bregua et al., 2018; Ganesh, 2021). Various stressor activated the HPA/HPI axis, which induced anxiety like behavior, reduced foraging and reproductive behaviors (Beitinger, 1990; Harris and Carr, 2016; Abreu et al., 2018; Filipsson et al., 2020). Corticotropin-releasing factor (CRF) and cortisol administration inhibited foraging behaviors, including food searching, prey capture, and food intake, in teleost fish (McCormick et al., 1998; Gregory and Wood, 1999; Bernier and Peter, 2001; Bernier et al., 2004). The HPA/HPI axis inhibited food intake may be due to slow energy support during digestion that was not efficient for emergent demands (Sapolsky et al., 2000). Elevated CRF and glucocorticoids inhibited food intake by stimulating leptin production and insulin releasing (Arase et al., 1988; Dallman et al., 1993; Michel and Cabanac, 1999). When animals are under internal unfavorable conditions, they activate and turn on the HPA/HPI axis and subsequently regulate the behavioral patterns to cope with an urgent stress.

### Effects of Abnormal Physiological Conditions on Fish Behaviors

Behaviors are integrative outcomes of physiological regulation in responding to external or internal stimuli, and they can also reflect abnormal or inefficient physiological and neural regulation of the disease. Behavioral tests are established and used in clinical medicine for a full diagnosis (Hort et al., 2010; Gossink et al., 2016; Perez-Lloret et al., 2021). Zebrafish and medaka have been used as models to investigate the etiology and pathophysiology of Parkinson’s disease (PD). Typically, there are two types of PD models: neurotoxin-induced and gene-based models to reduce dopaminergic neurons and dopamine release to mimic corresponding PD symptoms (Lam et al., 2005; McKinley et al., 2005; Matsui et al., 2012; Vaz et al., 2018; Najib et al., 2020; Razali et al., 2021). PD model fish also showed locomotion deficits, including a reduction in the swimming speed, an increase in the no-moving time, and impairment of touch sensations, which are similar to symptoms of PD patients (Lam et al., 2005; Milanese et al., 2012; Hughes et al., 2020; Robea et al., 2020; Wasel and Freeman, 2020). Thus, these behavioral characteristics may reflect motor function deficits of the central nervous system (CNS) and selective dopaminergic neuron losses.

The immune response may mediate behavioral responses (Dantzer, 2006). It was found that concentrations of cytokines of fish with high avoidance differed from those of low-avoidance fish in a novel-object test (Kirsten et al., 2018a). Bacterin-treated fish showed alterations of brain activities and lowered social preferences and exploratory behaviors toward novel objects (Kirsten et al., 2018b). Tilapia lake virus infection caused decreases in zebrafish locomotion and food intake, abnormal swimming patterns, and histopathological changes in the zebrafish brain (Mojzesz et al., 2021). This suggests that the immune system may mediate defensive responses at the behavioral level to minimize potential risks of infection and reallocate energy to healing. These cases mentioned previously reveal strong connections and high linkages between physiological states and behavioral changes, and behavioral response could reflect the current physiological states.

## Effects of Environmental Influences on Behavior

Compared to terrestrial animals, most of fish are ectotherms and live in water, so they may be more easily affected and more sensitive to environmental changes (Hansen et al., 2016; Olusanya and van Zyll de Jong, 2018). The physiological status of fish is directly and rapidly affected by environmental factors, including temperature, ions, gases, and chemicals (Lushchak and Bagnyukova, 2006; Valavanidis et al., 2006; Keen et al., 2017; Tong et al., 2020; Alfonso et al., 2021; Shih et al., 2021). Behavioral responses caused by changes in the physiological status have been widely published. Such studies suggested that environmental fluctuations may cause behavioral alterations through physiological regulation, and behavioral tests have high potential for assessing physiological states and environmental stimuli.

### Effects of Global Climate Changes on Fish Behaviors

Behavioral changes often point to underlying physiological alterations that can be used to evaluate ecological risks. Global climate change has attracted much attention recently, and warming and acidification are two very important issues among the multitude of ecosystem-level stressors (Byrne, 2011; IPCC, 2014).

Warm temperatures cause guppy schools to swim closer together and faster under a predator threat (Weetman et al., 1998; Weetman et al., 1999). This behavior might be due to the need for increased feeding at elevated temperatures and may be associated with an increased risk assessment of predators during feeding. In rainbow trout, higher temperatures increased the time spent feeding in risky, open habitats and increased mortality (Biro et al., 2007). Juvenile European sea bass undertake riskier behaviors such as reducing their shoaling cohesiveness, swimming higher above the bottom, and reducing the distance between the shoal and a predator at higher temperatures (Malavasi et al., 2013). Warm temperatures also decrease oxygen solubility and cause a rapid depletion of oxygen levels of fish (Verberk et al., 2011). Oxygen is crucial for metabolism, and the nervous system is very vulnerable to hypoxia. Acute hypoxia caused over 50% of deaths in adult zebrafish in 10 min, and surviving fish showed significant stress responses in the brain (increased cell proliferation and astrocyte numbers) and lower exploratory behaviors at 6 h after recovery (Braga et al., 2013; Lee et al., 2018). A lower responsiveness and impairment of directionality to a startling mechanical stimulus in the escape performance of grey mullet (*Liza aurata*) and European sea-bass (*Dicentrarchus labrax*) were found when exposed to progressive hypoxia (Lefrancois et al., 2005; Lefrançois and Domenici, 2006). To prevent the hypoxia damage, behavioral response may be activated upon hypoxia stress. Chemosensory neuroepithelial cells (NECs) located in primary gill filaments (Dunel-Erb et al., 1982) sensed oxygen level (Jonz et al., 2004) and initiated hyperventilation (Perry et al., 2009). Lower partial pressure of oxygen in water inhibited K^+^ permeability, elevated membrane potential, eventually activated voltage-gated Ca^2+^ channel and release neurotransmitter (possibly 5-HT or ACh) from NECs (Jonz et al., 2004; Shakarchi et al., 2013; Porteus et al., 2014; Jonz et al., 2015). Zebrafish larvae rapidly increased their body movements and beating of their pectoral fins to gain more oxygen within few second of a hypoxic stimulus (Erickstad et al., 2015). Low oxygen levels reduced the structure and size of schools to prevent an exacerbation of hypoxic conditions in herring (Domenici et al., 2002) and *C. auratus* (Israeli and Kimmel, 1996). Weakly electric fish (*Marcusenius victoriae* and *Petrocephalus degeni*) decreased the active acquisition of sensory information to save energy in a hypoxic condition (Ackerly et al., 2018; Clarke et al., 2020). Escape responses in fish are facilitated by anaerobically fueled muscles (Domenici and Blake, 1997; Marras et al., 2011; Marras et al., 2013); thus, the behavioral alteration is most probably due to a malfunctioning at the neurosensory level rather than to an impairment of muscle function (Domenici et al., 2007). Avoidance and energy-saving behavioral responses reflects current hypoxia challenges and can possibly be used to predict physiological condition.

Increased anthropogenic CO_2_ emissions not only elevate the temperature but also acidify aquatic environments. Water acidification may alter different sensory modalities, especially to olfactory system, which directly exposed and analyzed chemical composition of surrounding environment (Ache and Young, 2005; Briffa et al., 2012). For teleost, the sense of smell plays important roles in feeding and reproduction, predator avoidance. Indeed, many studies reported that water acidification affect olfactory mediated behavior in marine fish (Porteus et al., 2018; Velez et al., 2019). Juvenile reef fish were usually repelled by predator odor and chemical alarm cues (chemical compounds released from the damaged epidermal tissue); however, coral reef damselfish reared under elevated CO_2_ became attracted to predator odor (Dixson et al., 2010; Welch et al., 2014) and had reduced aversion to chemical alarm cues (Ferrari et al., 2011; Welch et al., 2014). Juvenile pink salmon reared in high-CO_2_ waters exhibited reduced electrophysiological responses to amino acids that are important for identifying natal rivers when they return from the ocean to spawn (Ou et al., 2015). Ocean acidification induced slight changes in Cl^−^ and/or HCO^3−^ gradients across membranes of neurons altered GABA_A_ receptor functions and cognition of fish (Evans et al., 2005; Nilsson et al., 2012). Elevated CO_2_ decreased the intensity of anti-predator behaviors in juvenile damselfish in response to visual predation risks (Ferrari et al., 2012). Californian rockfish and barramundi showed increased anxiety and sheltering behaviors under high CO_2_ levels (Hamilton et al., 2014; Rossi et al., 2015). These studies reveal the changes in olfactory and neural system functioning leads to inappropriate behavioral responses to environmental chemical stimuli.

### Effects of Anthropogenic Pollution on Fish Behaviors

Freshwater ecosystems such as rivers, lakes, and wetlands are vulnerable to human pollution (Dudgeon, 2019; Reid et al., 2019). Pesticides and herbicides used in agriculture can contaminate water through runoff from treated plants and soil (Aktar et al., 2009). Studies in India revealed that more than 90% of water and fish samples contained one and more often several pesticides (Kole et al., 2001). Studies of deltamethrin, an insecticide, found increased glutamate levels in the brains and led to hyperactive social behaviors in adult zebrafish (Lei et al., 2022). Fipronil causes neuronal hyperexcitation by inhibiting GABAergic neurotransmission and induces notochord degeneration and locomotor defects in zebrafish embryos and larvae (Tingle et al., 2003; Stehr et al., 2006). Moreover, a marked decrease of swimming endurance and anxiolytic effects were also found in fipronil-exposed adult zebrafish (Cuenca et al., 2022). The widely used herbicide, acetochlor, increased the time spent in the dark zone, suggesting promotion of anxiolytic behaviors (Huang et al., 2021). These behavioral changes caused by pesticide and herbicide exposure may lessen chances for survival of aquatic animals and threaten their populations as a whole.

Heavy metals from anthropic activities, such as the steel industry, mining activities, and smelters, are widely found in the environment and cause neurotoxicities (Squadrone et al., 2013; Zhong et al., 2018; Jiang et al., 2019). It was found that aluminum accumulates in the nervous system of fish and causes damage to nerve tissues, behavior, and cognition (Closset et al., 2021). Silver impairs social preferences, social recognition, learning, and memory in adult zebrafish (Fu et al., 2021). Cadmium induces CNS impairment and neuroinflammation and impairs social behaviors, escape behaviors, and predator responses in rainbow trout, sea bass, and zebrafish (Sloman et al., 2003; Blechinger et al., 2007; Faucher et al., 2008; Xu et al., 2022). Lead-exposed zebrafish exhibited decreased learning and altered color preferences (Bault et al., 2015; Xu et al., 2016). Mercury exposure causes Hg accumulation in the brain and an altered anxiety status, decreased foraging efficiency, and increased prey capture speed in seabream and fathead minnows (Grippo and Heath, 2003; Amlund et al., 2015; Pereira et al., 2016). Heavy metals caused imbalance between the reactive oxygen species (ROS) and the antioxidants, which increased oxidative stress and induced apoptosis (Karri et al., 2016). The damage on neurovascular system may lead to heavy metals pass through the blood–brain barrier and directly affected brain cells (Olung et al., 2021). In addition, heavy metals disturbed neurotransmission and caused cognition dysfunction in sublethal concentrations (Hayat et al., 2003; Toscano and Guilarte, 2005; Karri et al., 2016). Those studies demonstrated the neurotoxicity of heavy metals on behavioral level.

## Environmental Risk Assessments With Behavioral Monitoring

Behaviors are a type of functional physiological organization that enables animals to react and acclimate to complex environmental (external) and physiological (internal) stimuli (Hellou, 2011; Morton et al., 2016). Behavioral response with sublethal pollutant concentrations were reported to be fast and sensitive biomarkers for water quality, ecotoxicological and chemical risk assessments (Hellou, 2011; Melvin and Wilson, 2013; Pyle and Ford, 2017; Legradi et al., 2018; Xia et al., 2018). Some behavioral outcomes have been measured in contaminant-exposed fish, including swimming speeds, startle responses, prey capture abilities, learning, and memory, which related to the probability of escaping a predator or capturing prey for feeding (Legradi et al., 2018; Bownik and Wlodkowic, 2021; Fitzgerald et al., 2021). Considering the increasing numbers of environmental contaminants and their unknown neurotoxic potentials, identifying and improving neurotoxicity testing strategies and methods are important for eco-neurotoxicity assessments in the future (Pyle and Ford, 2017; Legradi et al., 2018; Xia et al., 2018; Fitzgerald et al., 2021).

## Concluding Remarks

In conclusion, behaviors are integrative outcomes of physiological responses in responding to internal or external stimuli and they also reflect abnormal physiological states (Supplementary Table S1). Thus, it is possible to implicate the alteration of behavioral patterns for environmental and physiological assessments.
