# Supplementary material for: Fish Behavior as a Neural Proxy to Reveal Physiological States
Source: Front Physiol. 2022 Jul 13;13:937432. doi: 10.3389/fphys.2022.937432 (PMC9326089; doi:10.3389/fphys.2022.937432)
Supplement: Supplementary file 1 [file DataSheet1.PDF]

Supplementary Table 1 Effects of internal states and external stimuli on fish behaviors

	**Stimulus**	**Species**	**Behavioral effects**	**Cited references**
**Internal**				
	Loss of buccal water	Mudskippers (*Periophthalmus modestus*)	Drink and refill water	(Katayama et al., 2018)
	Starvation	Common goldfish (*Carassius auratus*)	Move to risky, open area for food	(Katz et al., 2013)
	Starvation	Common goldfish (*Carassius auratus*)	Leave refuge for food	(Balaban-Feld et al., 2019)
	Starvation	Zebrafish (*Danio rerio*)	Potentiate Hb to IPN winner pathway and win in social conflict	(Nakajo et al., 2020)
	AgRP1	Zebrafish (*Danio rerio*)	Increase food consumption	(Shainer et al., 2019)
	Cortisol	Goldfish (*Carassius auratus*)	Decrease food intake and growth rate	(Bernier et al., 2004)
	Cortisol	Rainbow trout (*Oncorhynchus mykiss)*	Inhibit food intake	(Gregory and Wood, 1999)
	Physical stressors	Atlantic salmon (*Salmo salar*)	Inhibit food intake	(McCormick et al., 1998)
	Lacking both β- and γ1-synucleins	Zebrafish (*Danio rerio*)	Decrease spontaneous motor activity	(Milanese et al., 2012)

	1-methyl-4-phenyl-1,2,3,6-tetrahydropyridine (MPTP)	Zebrafish (*Danio rerio*)	Impair swimming responses to touch stimulus	(Lam et al., 2005)
	PARK7 gene knockout	Zebrafish (*Danio rerio*)	Decrease moving duration and spontaneous tail beating frequency	(Hughes et al., 2020)
	Aeromonoas hydrophila bacterin	Zebrafish (*Danio rerio*)	Decrease social preference and exploratory behavior	(Kirsten et al., 2018b)
	Low expression of INF-γ	Zebrafish (*Danio rerio*)	Decrease social preference	(Kirsten et al., 2018a)
	Tilapia lake virus	Zebrafish (*Danio rerio*)	Decrease locomotion activity and time spent in upper zone of tank	(Mojzesz et al., 2021)
**External**				
	Temperature	European sea bass (*Dicentrarchus labrax*)	Show highest shoal cohesiveness at 22°C; Prolong antipredator	(Malavasi et al., 2013)

			response duration at 18°C than at 22°C and 26°C	
	Temperature	Guppy (*Poecilia reticulata*)	Choose to shoal with the smaller group when the predator was absent	(Weetman et al., 1999)
	Temperature	Guppy (*Poecilia reticulata*)	Become close schooling pattern at 26°C than at 22°C	(Weetman et al., 1998)
	Temperature	Rainbow trout (*Oncorhynchus mykiss)*	Increase feeding frequency, resulted in greater vulnerability to predators	(Biro et al., 2007)
	Hypoxia	Herring (*Clupea harengus*)	Change schooling pattern	(Domenici et al., 2002)
	Hypoxia	Golden grey mullet (*Liza aurata)*	Disturb the orientation of escape behavior	(Lefrancois et al., 2005)
	Hypoxia	European sea bass (*Dicentrarchus labrax*)	Disturb the orientation of	(Lefrançois and

			escape behavior	Domenici, 2006)
	Hypoxia	Zebrafish (*Danio rerio*)	Decrease locomotion activity and exploratory behavior	(Braga et al., 2013)
	Hypoxia	Zebrafish (*Danio rerio*)	Impair learning and memory ability	(Lee et al., 2018)
	Hypoxia	Zebrafish (*Danio rerio*)	Increase pectoral fin beats and body movement	(Erickstad et al., 2015)
	Hypoxia	Zebrafish (*Danio rerio)*	Increase ventilation frequency	(Shakarchi et al., 2013)
	Hypoxia	Weakly electric fish (*Petrocephalus degeni*)	Decrease sensory information acquisition	(Clarke et al., 2020)
	Hypoxia	African mormyrid fish (*Marcusenius victoriae*)	Decrease sensory information acquisition	(Ackerly et al., 2018)
	CO_2	Barramundi (*Lates calcarifer)*	Reduce the swimming speeds and increase the	(Rossi et al., 2015)

			duration in shelter	
	CO_2	Californian rockfish (*Sebastes diploproa*)	Increase duration in dark zone	(Hamilton et al., 2014)
	CO_2	Damselfish (*Pomacentrus chrysurus)*	Lose innate avoidance of chemical alarm cue; Move further away from the reef	(Ferrari et al., 2011)
	CO_2	Damselfish (*Pomacentrus amboinensis)*	Reduce antipredator responses	(Ferrari et al., 2012)
	CO_2	Damselfish (*Neopomacentrus azysron*)	Disrupted behavioral lateralization	(Nilsson et al., 2012)
	CO_2	Pink salmon (*Oncorhynchus gorbuscha*)	Decrease function of olfactory epithelium	(Ou et al., 2015)
	CO_2	European sea bass (*Dicentrarchus labrax*)	decreases the amplitude of the olfactory response	(Porteus et al., 2018)
	CO_2	Gilthead seabream (*Sparus aurata*)		(Velez et al., 2019)
	CO_2	Orange clownfish (*Amphiprion percula*)	Be attracted to the smell of predators	(Dixson et al., 2010)
	CO_2	Spiny damselfish (Acanthochromis polyacanthus)	Lose innate avoidance of	(Welch et al., 2014)

			chemical alarm cue	
	Silver	Zebrafish (*Danio rerio*)	Impair social behavior and fear learning	(Fu et al., 2021)
	Cadmium	Zebrafish (*Danio rerio*)	Reduce the response to environmental signals	(Xu et al., 2022)
	Cadmium	Rainbow trout (*Oncorhynchus mykiss*)	Form the dominance-subordinate hierarchies faster	(Sloman et al., 2003)
	Cadmium	Sea bass (*Dicentrarchus labrax)*	Decrease the response to lateral system stimulations	(Faucher et al., 2008)
	Cadmium	Zebrafish (*Danio rerio*)	Impair olfactory-dependent predator avoidance behaviors	(Blechinger et al., 2007)
	Lead	Zebrafish (*Danio rerio*)	Alter color preferences	(Bault et al., 2015)
	Lead	Zebrafish (*Danio rerio*)	Impair fear learning behavior	(Xu et al., 2016)

	Mercury	White seabream (*Diplodus sargus*)	Reduce the swimming velocity	(Pereira et al., 2016)
	Mercury	Fathead minnows (*Pimephales promelas*)	Impair foraging efficiency and capture speed	(Grippo and Heath, 2003)
